# Renal collision tumours: three additional case reports

**DOI:** 10.1186/s12894-022-01063-y

**Published:** 2022-07-23

**Authors:** Valère Belle Mbou, Florian Sanglier, Julia Pestre-Munier, Aurélien Descazeaud, François Labrousse

**Affiliations:** 1 Service d’Anatomie pathologique, CHU de Limoges, 2 avenue Martin Luther King Limoges cedex, 87042 Limoges, France; 2 Service de Radiologie et imagerie médicale, CHU de Limoges, 2 avenue Martin Luther King Limoges cedex, 87042 Limoges, France; 3 Service d’Oncologie médicale, CHU de Limoges, 2 avenue Martin Luther King Limoges cedex, 87042 Limoges, France; 4 Service de Chirurgie urologique et andrologie, CHU de Limoges, 2 avenue Martin Luther King Limoges cedex, 87042 Limoges, France

**Keywords:** Tumour, Kidney, Histology, Prognostic, Histogenesis

## Abstract

**Background:**

Multiple kidney tumours are frequently seen in hereditary syndromes and familial diseases. Renal collision tumours (RCT) are characterized by the simultaneous existence of different and unrelated tumour types within the same location in the kidney, forming a single, heterogenous lesion. RCT are uncommon histological entities with distinctive features. The most frequent subtypes include clear cell renal cell carcinoma (CCRCC), papillary renal cell carcinoma (PRCC), chromophobe renal cell carcinoma (CRCC), and collecting duct carcinoma (CDC).

**Case presentation:**

Here, we report three sporadic cases of RCT successfully treated by nephrectomy and confirmed by histological analysis. The first case was of a 64-year-old man diagnosed with RCT composed of a stage 2 nucleolar grade 3 CCRCC and a stage 1a nucleolar grade 2 type 1 PRCC. The second case was of a 68-year-old woman diagnosed with a combined nucleolar grade 2 type 1 PRCC and an angiomyolipoma (non-assessed stage), while the third case was of a 59-year-old woman diagnosed with a combined stage 1a nucleolar grade 3 CCRCC and a stage 1b CDC.

**Conclusions:**

Due to the rarity of RCT, there are no standard guidelines for their management. Hence, the prognosis is considered to be associated with the most aggressive component, possibly the tumour with the highest nucleolar grade and stage. The histogenesis of RCT remains debated, and increase in knowledge regarding this can help enable the development of targeted therapies for advanced or metastatic tumours.

## Background

Renal cell carcinoma (RCC) is a heterogeneous group of tumours representing ~ 2–4% of all adult cancers [1]. The risk factors for developing kidney tumours include smoking, obesity, high blood pressure, and exposure to certain toxic substances. The presence of multiple renal tumours is more frequently observed in people with hereditary conditions such as von Hippel-Lindau disease, Birt-Hogg-Dubé syndrome, hereditary papillary renal cell carcinomas (PRCC), hereditary leiomyomatosis, and tuberous sclerosis of Bourneville (TSB). Multifocality has also been observed in acquired cystic kidney disease and renal oncocytomatosis [2]. The incidence of sporadic multifocal renal tumours has been shown to vary between 4 and 20% at the time of diagnosis [3]. Tumours that are composed of a combination of different histological types in the same location within an organ are referred to as collision tumours, which have been reported to occur in several organs [4]. Here, we present three rare and sporadic cases of renal collision tumors (RCT), one involving clear cell renal cell carcinoma (CCRCC) and PRCC in a 64-year-old man, the second involving PRCC and angiomyolipoma (AML) in a 68-year-old woman, and the third involving CCRCC and duct collecting carcinoma (CDC) in a 59-year-old woman. All three cases were successfully treated by nephrectomy.

### Case presentation

#### Case report 1

**Fig. 1:**
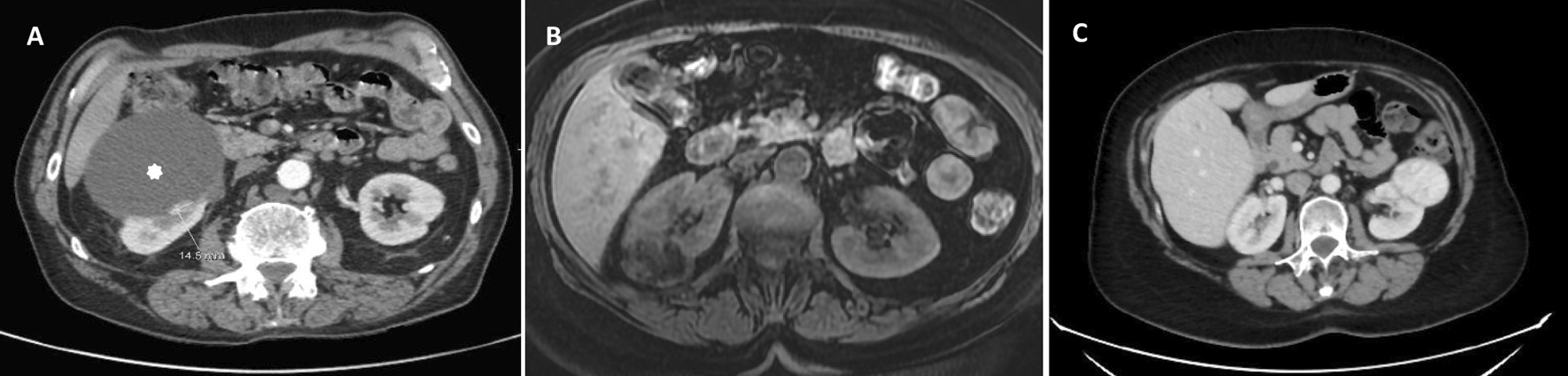
Abdominal computed tomography scan showing a Bosniak 4 cystic lesion (white star) and a fleshy lesion (white line) of right kidney in Case 1 (**A**), a right mid-renal lesion with fat and tissular components in Case 2 (**B**) and a left kidney mass in Case 3 (**C**)

A 64-year-old man with a venous thromboembolic disease (heterozygous factor V mutation) underwent regular follow-ups. He was a uranium miner, had smoked for 50 years, and was an alcoholic. Thoraco-abdominopelvic computed tomography (CT) revealed a 100-mm lower-polar right renal cyst, classified as Bosniak IV with a 20-mm posterosuperior wall tissue nodule (Fig. 1A). A kidney biopsy showed a nucleolar grade 3 CCRCC combined with a nucleolar grade 2 type 1 PRCC.

**Fig. 2:**
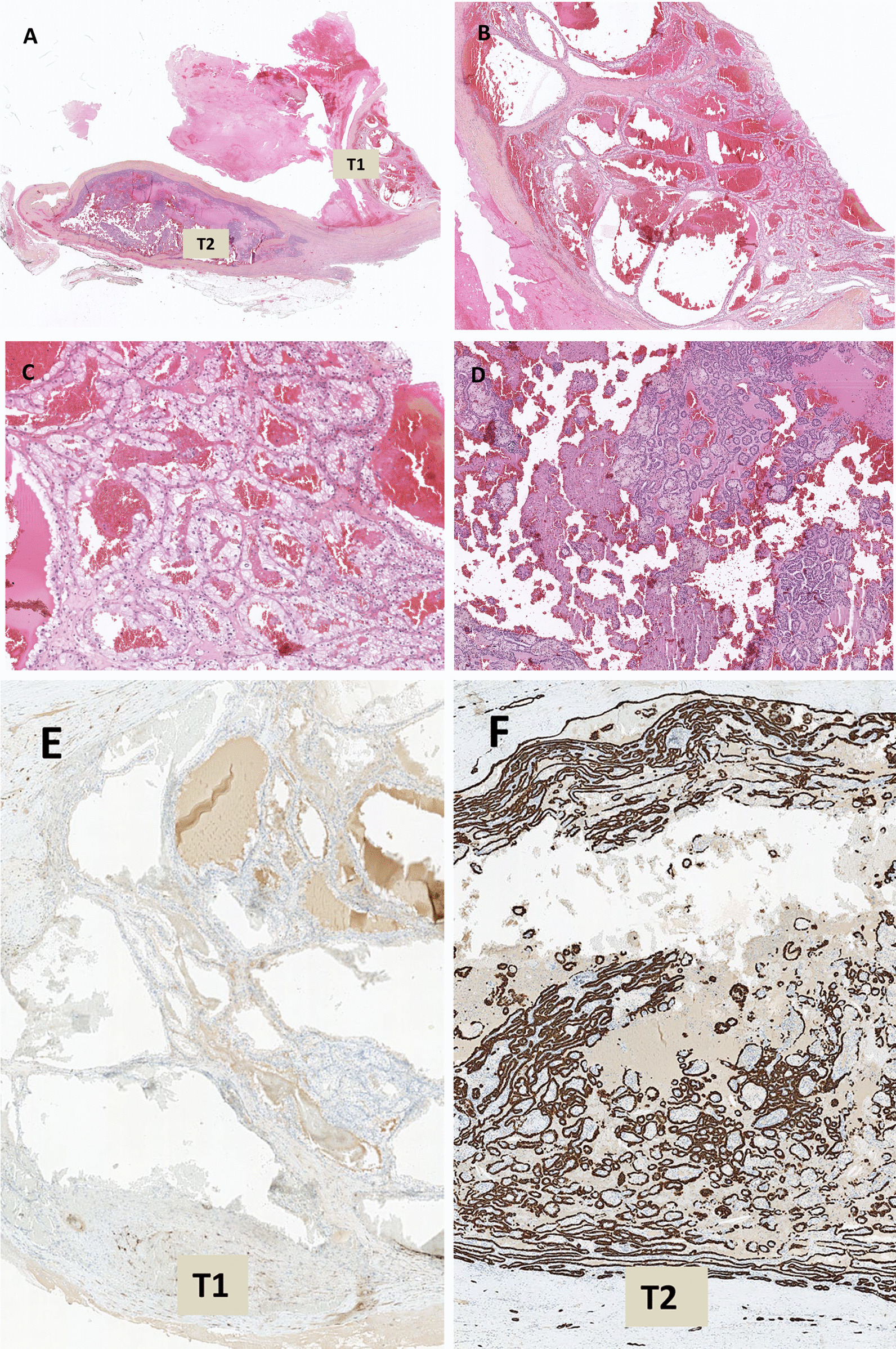

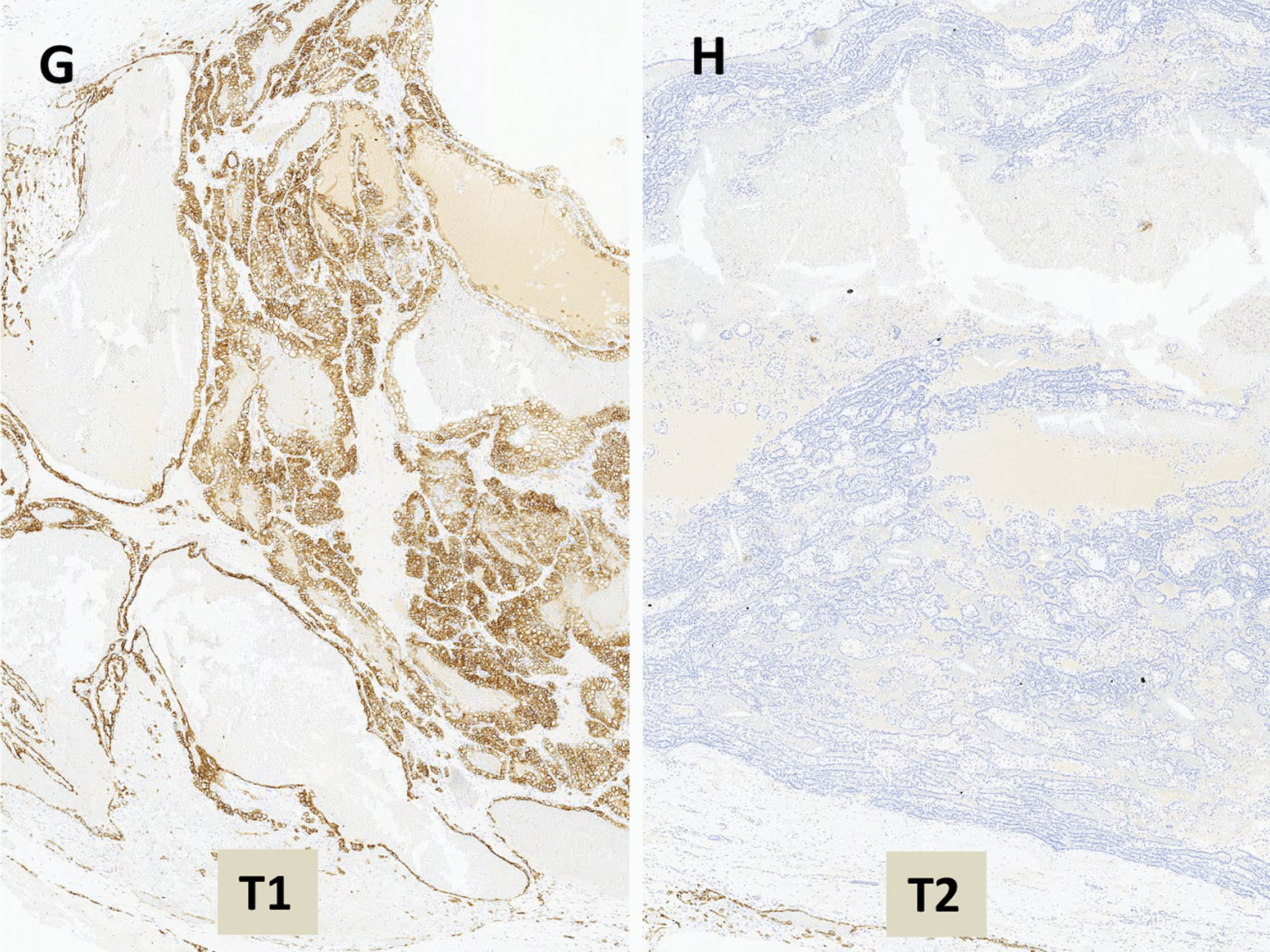
Histologic features of collision renal tumour associating CCRCC and PRCC (hematoxylin and eosin, immunophenotype). Renal collision tumour combining two tumours: CCRCC «T1» and PRCC «T2» (**A**). Tumour T1 is composed of cystic and tubular pattern of clear cell carcinoma (**B**). Tumour T1 is constituted with atypical clear cell (**C**). Tumour T2 is arranged in papillary pattern (**D**). CK7 immunohistochemistry is negative in T1 (**E**) and positive in T2 (**F**) whereas CA-IX immunohistochemestry is positive in T1 (**G**) and negative in T2 (**H**)

The macroscopic, microscopic, and immunohistochemical features of the patient’s sample are described in Table 1 and Fig. 2.

**Table 1:** Macroscopic, microscopic and immunohistochemical features of the samples from the three patients

	Case report 1	Case report 2	Case report 3
Macroscopic features	Partial nephrectomy specimen of 596 g and 160 × 130 × 70 mm was 30% necrotic and composed of a cystic lesion (T1) of 110 × 110 × 70 mm with solid, friable, grey-yellow content. Within the cystic wall, there was a reasonably well-limited nodular lesion (T2) of 20 × 15 × 15 mm, which was firm and grey-white. The non-tumorous renal parenchyma was 50 × 20 × 18 mm in size.	Surgical specimen of 86 g was fragmented. Its grouping made it possible to vaguely reconstitute a 70 × 35 × 15 mm renal fragment that was surrounded by adipose tissue and contained a renal lesion, which was ~ 38 mm in diameter, firm, and greyish-white in colour, with necrotic and cystic zones.	Surgical specimen (395 g) contained a kidney measuring 11 × 8.5 × 4.5 cm. A well-limited mid-renal tumour of 70 × 65 mm was observed, which was non-encapsulated and fleshy, had a yellow to pale grey cut surface, and did not show necrosis or thrombus in the renal vein.
Microscopic features	Two tumours, T1 and T2 were quite distinct microscopically and separated by fibrous tissue (Fig. 2A). Tumour (T1) was a malignant neoplasm with tubulocystic architecture (Fig. 2B). The cells were large, with clear cytoplasm and irregular nuclei, and contained nucleoli that were visible at 100 × magnification (Fig. 2C). Tumour (T2) was composed of papillae formed by fibrovascular cores that often-contained foamy macrophages and sometimes psammoma bodies (Fig. 2D). These papillae were bordered by cubic cells with weakly eosinophilic cytoplasm and rounded nuclei that contained nucleoli perceptible at 400 × magnification. No tumour embolus was observed. Surgical borders were intact.	Tumour (T1) represented two-thirds of the tumour volume and was inconsistently separated from another tumour (T2) by a thin fibrous septum (Fig. 3). T1 tumour produced papillary features within a fine fibrous stroma reaction. The papillae sometimes contained foamy macrophages in their axes and were bordered by a single layer of eosinophilic cubic cells with irregular nuclei and nucleoli visible at 100 × magnification. No tumour emboli were present. T2 tumour was composite and consisted of short bundles of spindle cells without nuclear atypia or epithelioid differentiation, vessels with thick and fibrous walls, and lobules of mature adipocytes. Perinephric flat was devoid of tumour invasion.	There was no evidence of fibrous septa between T1 and T2 tumours (Fig. 4A). T1 tumour consisted of large, clarified cells, with irregular nuclei containing nucleoli visible at 100 × magnification, arranged in a solid and acinar pattern. T2 tumour was made of atypical eosinophilic cells, displaying tubes, papillae, and solid clusters. No tumour embolus or extension in the perinephric flat was present.
Immunohistochemical features	Tumour cells of the first lesion (T1) were positive for CD10 and CA-IX, and negative for CK7 and P504S. In contrast, tumour cells of the second lesion (T2) were positive for CK7 and P504S, and negative for CD10 and CA-IX (Figs. 2E-H).	Tumour cells of T1 were immunoreactive with CK7 but not with CD10 or CA-IX. Spindle cells of T2 expressed smooth muscle actin, caldesmon, and HMB-45.	T1 tumour cells were positive for Vimentin, CA-IX, CD10 and PAX8, negative for CK7, P63 and CD117. In contrast, the T2 tumour cells were positive for Vimentin, CA-IX, CD117 and PAX8, negative for CK7, CD10 and P63 (Figs. 4B-C).

The histological diagnosis of a RCT that combined a nucleolar grade 3 CCRCC measuring 110 mm (stage pT2; UICC 2017) and a nucleolar grade 2 type 1 PRCC measuring 16 mm (stage pT1a) was made, and an R0 excision was performed.

Twenty months into the clinical course, the patient is well and shows no evidence of tumour recurrence or metastatic lesions.

#### Case report 2

A 68-year-old woman underwent right partial nephrectomy for a right kidney tumour discovered during a check-up for pelvic trauma from a horse-riding accident. Magnetic resonance imaging revealed a heterogeneous 40 mm mid-renal mass with fat and tissue elements, which showed heterogeneous enhancement after gadolinium injection (Fig. 1B).

**Fig. 3:**
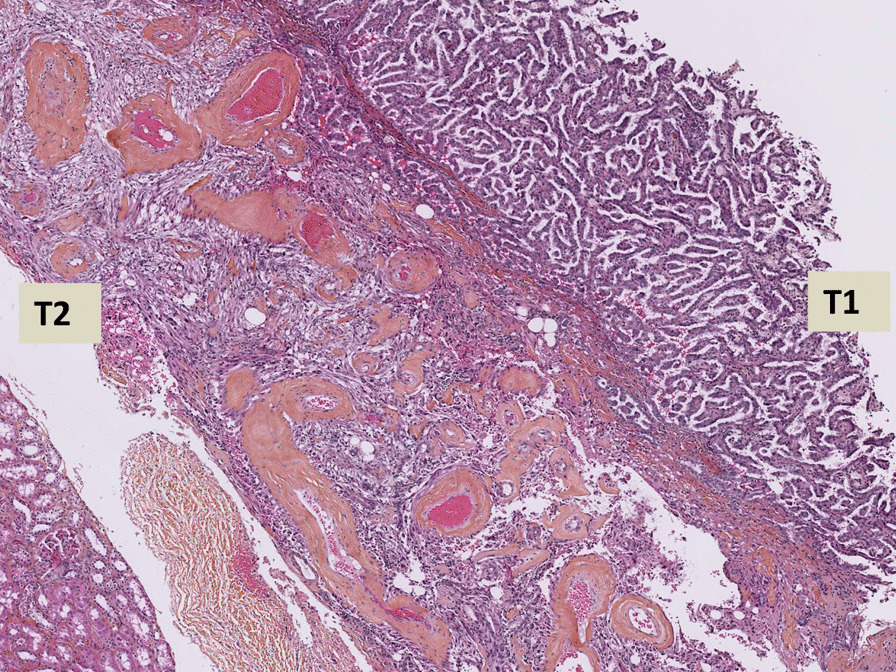
Histologic features (hematoxylin and eosin) of renal collision tumour combining PRCC «T1» and AML «T2»

The macroscopic, microscopic, and immunohistochemical features of the patient’s sample are described in Table 1 and Fig. 3.

The histological diagnosis of a RCT with nucleolar grade 3 type 1 PRCC and AML was made. The tumour size and surgical margins could not be determined because the partial nephrectomy piece was fragmented.

The clinical course of the patient was unremarkable, and repeat CT did not show any recurrence after fourteen months of follow-up.

#### Case report 3

A 59-year-old woman was referred by her rheumatologist after a CT scan revealed a left kidney mass of 65 mm (Fig. 1C) during a chronic biological inflammatory syndrome check-up.

A kidney biopsy revealed a nucleolar grade 3 CCRCC. The patient underwent an enlarged left nephrectomy laparoscopically.

**Fig. 4:**
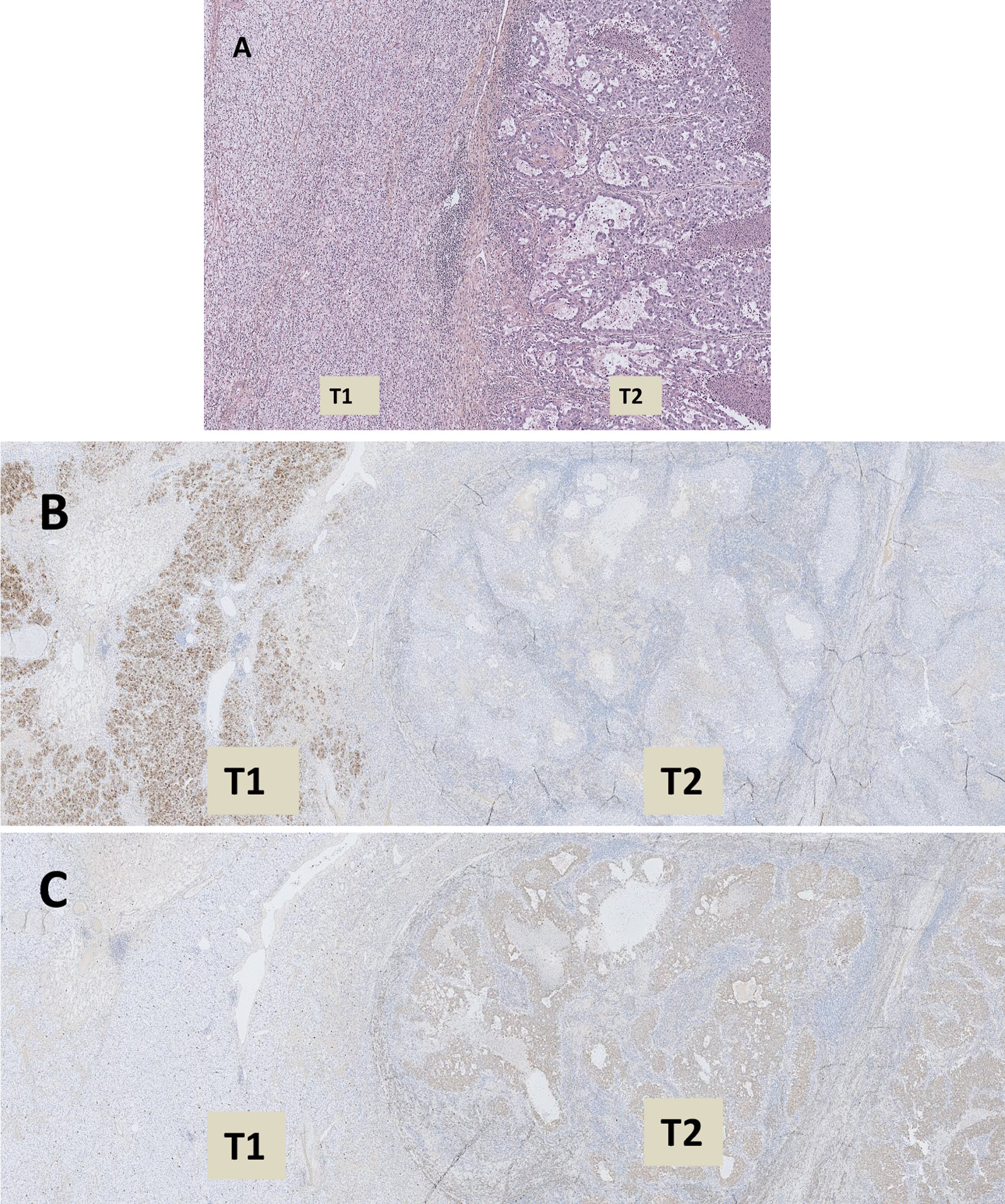
Histologic features of renal collision tumour associating CCRCC «T1» and CDC «T2» (hematoxylin and eosin, immunophenotype). Renal collision tumour combining two tumours: CCRCC «T1» and CDC «T2» (**A**). CD10 immunohistochemistry is positive in T1 and negative in T2 (**B**) whereas CD117 immunohistochemestry is negative in T1 and positive in T2 (**C**)

The macroscopic, microscopic, and immunohistochemical features of the patient’s sample are described in Table 1 and Fig. 4.

The histological diagnosis revealed a RCT consisting of a nucleolar grade 3 CCRCC measuring 28 mm (stage pT1a) and a CDC measuring 42 mm (stage pT1b).

At 12 months, the clinical evaluation was satisfactory and there was no recurrence.

## Discussion and conclusions

The term ‘collision tumour’ refers to a phenomenon in which two or more distinct and unrelated tumour types are present at the same location, forming a single lesion. These tumours are usually limited and do not commonly undergo tumour changes [5]. However, it is challenging to distinguish between collision and composite tumours among the renal tumour cases cited in the literature. Similar to collision tumours, composite tumours consist of different tumours in a single lesion, but they do not have clear limits and usually undergo tumour changes. Synchronous tumours, however, comprise different tumour types in different locations within the same organ [6].

According to previous studies, collision tumours occur in many organs, including the lungs, digestive tract, and ovaries [4, 7, 8]. However, they rarely develop in the kidneys; most reported cases are unique ones [9]. Specifically, these tumours contain either two malignant components, as seen in Cases 1 and 3, or one malignant and one benign component, as seen in Case 2 [10, 11]. A review of 14 RCT cases with two previously published malignant components of renal origin along with our two cases (Cases 1 and 3) are presented in Table 2 [9, 11–23].

**Table 2:** Reported cases of collision renal tumours combining two malignant components

Cases [references]	Sex	Age (years)	Symptoms	Tumour 1	Tumour 2	Follow-up period
1- Anani et al. [9]	Male	81	Not available	CRCC	PRCC	Not available
2- Zhang et al. [11]	Female	63	Hematuria	PRCC	CRCC	NED at 20 months
3- Burch-Smith et al. [12]	Female	67	Abdominal pain	CCRCC	CDC	Metastases 5 months post-nephrectomy
4- Elian & Lam [13]	Male	66	Hematuria	PRCC	RMC	Not available
5- Kawano et al. [14]	Female	64	Abdominal pain, fever	CRCC	CDC	Metastases 8 months post-nephrectomy
6- Bartos et al. [15]	Male	70	Not available	CCRCC	PRCC	NED at 36 months
7- Matei et al. [16]	Male	70	Abdominal pain, hematuria	CDC	PRCC	NED at 57 months
8- Cho et al. [17]	Male	24	Accidental finding	CCRCC	CDC	Not available
9- Gong et al. [18]	Male	72	Weight loss, asthenia, anemia	CRCC	CDC	Not available
10- Roehrl et al. [19]	Female	65	Accidental finding	CRCC	PRCC	NED at 4 months
11- Moe et al. [20]	Male	51	Accidental finding	PRCC	CCRCC	Not available
12- Salazar-Mejia et al. [21]	Male	47	Dry cough, weight loss	CCRCC	CDC	NED at 12 months
13- Lamprou et al. [22]	Male	68	Accidental Finding	CCRCC	PRCC	Not available
14- Compérat [23]	Female	67	Accidental finding	CCRCC	PRCC	Not available
Present Case report 1	Male	64	Accidental finding	CCRCC	PRCC	NED at 20 months
Present Case report 3	Female	59	Accidental finding	CCRCC	CDC	NED at 12 months

*CRCC* chromophobe renal cell carcinoma; *PRCC* papillary renal cell carcinoma; *CDC* collecting duct carcinoma; *CCRCC* clear cell renal cell carcinoma; *RMC* renal medullary carcinoma; *NED* no evidence of disease

Studies have shown that RCC accounts for 2%–4% of adult malignancies and is predominant in men [1]. This was confirmed by the data collected in our study (Table 2), consisting of data from 10 male patients and 6 female patients aged 24–81 years (mean: 62 years). Moreover, the four main types of RCC (CCRCC, PRCC, chromophobe RCC, and CDC) are listed in Table 2. The most important associations were found in CCRCC and PRCC (5/16 cases), followed by chromophobe RCC and PRCC (3/16 cases). However, it was unfair to draw a conclusion regarding sex distribution and dominance of association due to the limited number of patients. Similar to classic renal tumours, collision tumours are usually discovered either incidentally or due to symptoms related to tumour expansion. Furthermore, smoking and exposure to industrial toxicants, as seen in Case 1, are risk factors associated with renal tumour development.

AML is considered the most common benign renal tumour, with a prevalence of 0.2%–0.6% of adult renal tumours and a female predominance. It is a mesenchymal tumour that belongs to the perivascular epithelioid cell group of tumours, which has been observed in nearly three-quarters of patients with certain hereditary factors such as TSB [2]. In Case 2, no hereditary or familial risk factors were identified. Similarly, renal AML in previous studies also appeared sporadically, but their association with other renal tumours was not well documented. Particularly, in a series of 36 collated cases of RCT associated with AML and renal cell tumours, Rafael et al. reported 25 sporadic cases and 11 TSB-related cases with mean ages of 59 and 53 years, respectively [24]. In this series, the association between AML and PRCC was found in only two of the 25 sporadic cases.

Regarding imaging appearance, collision tumours generally manifested atypical characteristics that offer limited information in the diagnostic process. In Case 1, CT was effective, consistent with the case reported by Goyal et al. [25]. The existence of possible collision tumours can be revealed on imaging when the capsule delimiting the two tumour components is well defined. However, when this border is not radiologically visible, as seen in Case 3 or in the cases reported by Gaeta et al. and McCroskey et al., radiological investigations may only show one mass [26, 27]. Therefore, it is essential to macroscopically sample the periphery of any renal mass to avoid misdiagnosis of any tumour association.

Recently, the interest in renal biopsy has re-emerged, especially in the management of small (less than or equal to 3 cm) renal solid masses [28]. Biopsy indications for small tumours, which has increasingly been discovered by imaging techniques, should be discussed during multidisciplinary conferences to establish a diagnosis that would guide the choice of management strategies. Although fine-needle biopsy can usually correctly classify three-fourths of classic renal tumours, renal biopsy may be more informative in cases of collision tumours if the biopsy fragments involve different tumour contents, as seen in Case 1 and in contrast to Case 3. So, the diagnosis frequency of collision renal masses obtained from percutaneous biopsy is not well documented. To our knowledge, the diagnosis of collision tumour made from percutaneous biopsies in case 1 is the first described in the English literature.

Despite the use of tumour biopsy, it can be challenging to analyse samples with two different co-existing histological types. A CCRCC and PRCC collision tumour (as seen in Case 1), in particular, must be distinguished morphologically from clear cell papillary renal cell carcinoma (CCPRCC), which is typically a low-grade renal carcinoma with histological aspects of both CCRCC and PRCC and was first described in 2006 [29]. CCPRCC displays a papillary, tubular, solid, or acinar architecture, with cells containing atypical nuclei that are polarised away from the basement membrane, creating characteristic sub-nuclear vacuoles such as the secretory endometrium [30]. Additionally, the axes of the papillae do not contain foamy macrophages or psammoma bodies, and the stroma occasionally exhibits mixed muscle fibre metaplasia. The tumour cells are positive for CA-IX and CK7 and negative for CD10 and P504S [30].

Notably, the prognosis of collision tumours is dependent on the nucleolar grade and RCC stage. In Case 3, the prognosis depended on both tumours as they were both high grade and Stage 1, whereas in Case 2, the prognosis of the tumour depended on PRCC since AML is a benign tumour. Furthermore, with the absence of tumour extension in the peri-renal adipose tissue in Case 2, complete tumour excision can be extrapolated. For collision tumours, it is essential to evaluate the relative share of the associated components, providing a histological type and stage for each. Thus, the prognosis is most likely related to the most aggressive component with the highest nucleolar grade and stage; however, there is no consensus on the clinical impact of CRT. Therefore, the treatment and follow-up of patients with CRT tumour have been based on standard guidelines for classic renal tumours [31].

The origin of the association between different tumour types in collision tumours is unclear. Therefore, different hypotheses have been proposed to explain its histogenesis. First, there may be two distinct cell lines proliferating simultaneously after a common oncogenic stimulus from the microenvironment, thereby causing two tumours with different phenotypes [12]. Second, the first tumour could alter the organ’s microenvironment, causing the second tumour to have a different phenotype [13]. Third, common precursor stem cells can differentiate into two different lineages and, therefore, cause two distinct tumours [14, 15]. Lastly, the appearance of two distinct tumours in the same anatomical site could have arisen accidentally after different oncogenic stimuli [32]. In Case 1, the two tumours, CCRCC and PRCC, had a common origin from the proximal tubule cells, thus pointing to the third hypothesis. In contrast, since AML originates from perivascular epithelioid cells different from PRCC, the histogenesis of Case 2 could be attributed to the second or fourth hypothesis. Moreover, since CCRCC originates from proximal tubule cells and CDC originates from distal tubule cells, the histogenesis of Case 3 would be similar to that of Case 2. Therefore, in-depth genomic studies on representative samples of collision tumours are fundamental for the elucidation of the somatic mutations responsible for these renal entities. Knowledge on the histogenesis mechanism of collision tumours can enable the development of targeted therapies for advanced or metastatic tumours.

In conclusion, collision tumours, which are composed of two distinct types of renal cell carcinoma, are rare and are usually discovered accidentally or due to symptoms related to tumour expansion. Histological diagnosis of biopsy specimens is challenging and requires exhaustive sampling of all associated tumour components. As the clinical impact of this entity has not been established due to its rarity, its management is based on standard guidelines for classic renal tumours. The histogenesis of such tumours is controversial, and its prognosis depends on the nucleolar grade and/or stage of the most aggressive component.

## Data Availability

Yes. The data can be found/requested from Limoges hospital archives. They can be consulted after making the request to the Research and Innovation Department (e-mail: abdeslam.bentaleb@chu-limoges.fr) specifying the title of case report, the authors and the ethics committee registration number (445–2021-101).
